# Importance of Increasing Modifiable Risk Factors Knowledge on Alzheimer’s Disease Among Community Pharmacists and General Practitioners in Spain

**DOI:** 10.3389/fphar.2019.00860

**Published:** 2019-08-14

**Authors:** Mónica Alacreu, Juan Pardo, María Azorín, María Teresa Climent, Vicente Gasull, Lucrecia Moreno

**Affiliations:** ^1^Embedded Systems and Artificial Intelligence Group (ESAI), Universidad CEU Cardenal Herrera, Valencia, Spain; ^2^Department of Pharmacy, Universidad CEU Cardenal Herrera, Valencia, Spain; ^3^Spanish Society of Family and Community Pharmacy (SEFAC), Valencia, Spain; ^4^Spanish Society of Primary Care Physicians (SEMERGEN), Valencia, Spain

**Keywords:** risk factors, Alzheimer’s disease, knowledge scale, community pharmacist knowledge, general practitioner knowledge, ADKS

## Abstract

Community pharmacists and general practitioners have daily contact with patients with Alzheimer’s disease (AD) but the number of positive cases constantly increases every day. Thus, the aim of this research is to describe the level of AD knowledge among community pharmacists and general practitioners in Spain, in order to see where the biggest gaps in the knowledge are. Therefore, a cross-sectional study has been carried out, using the Alzheimer’s disease knowledge survey (ADKS), among members of the Spanish Society of Primary Care Physicians and the Spanish Society of Family and Community Pharmacy to report the differences in AD knowledge in both professional collectives. The ADKS has been responded by 578 community pharmacists and 104 general practitioners and consists of a battery of 30 questions, whose possible answers are true or false. It assesses the AD knowledge in seven areas (impact on the disease, risk factors, course of the disease, diagnosis, care, treatment and symptoms). Results indicate that Spanish pharmacists and general practitioners have a high personal knowledge of AD, nevertheless, it is not associated with greater awareness. Both scored above 80% at the categories: diagnostic, treatment and symptoms. However, lower knowledge level (60% of correct answers) was found in those related to risk factors, such as the ignorance about hypercholesterolemia or hypertension as risk factors for the disease. Community pharmacists are already acting to control cardiovascular risk factors, but a wider knowledge of the relationship of these factors to AD is needed to act against these silent risk factors. Thus, pharmacists may also be involved in the management of AD that includes recognizing early symptoms for early detection of cognitive impairment. Hence, knowledge about risk factors is very important in developing this expanding role.

## Introduction

The aging of the world population is a reality. The reduction in mortality rates make the European Union the geographic area with the highest life expectancy of 83 years (Foreman et al., 2018; OECD, 2017). In fact, it is expected to increase to 85 years by 2040; nevertheless, the prevalence of chronic diseases such as dementia remains longer and in a greater proportion in the population (WHO, 2007; WHO, 2017; Martínez‐Lage et al., 2018).

Around 50 million people suffer from dementia worldwide and it is expected to rise to 75 million in 2030 and 132 million in 2050. Alzheimer’s disease (AD) is the most common type of dementia, corresponding to 60–80% of the cases. Moreover, it affects more than one million people to whom the undiagnosed cases should be added and that perturbs the lives of 4.5 million people among patients and carers (Prince et al., 2015).

Spain is one of the countries in the European Union that has the highest prevalence of dementia, between 5 and 8% of those over 60 years old a pattern is presented, accompanied by age and doubling every 5 years. The prevalence indicates a percentage of 5.5–5.8% in those over 65 and 8.5–9.4% in those over 70 years old. Furthermore, in 30 years time, 30% of the population would suffer from AD (Garre-Olmo, 2018).

Alzheimer’s disease has a relevant social and economic impact according to the evolutionary phase of the pathology, corresponding to medical costs, attention provided, training of caregivers, among others. It is estimated that the cost per patient per year is around 31.000 euros. It is also one of the main causes of disability and dependency, being responsible for 11.9% of years lived with disabilities (WHO, 2017). Additionally, it is also one of the main national and worldwide causes of death. In 2016 in Spain, of 410.611 deaths, 14.793 were because of AD, with 4.370 men and 10.423 women (INE, 2016).

Thus, early diagnosis would allow quick access to the appropriate treatment to be more beneficial, access to care services, and to reduce the psychological burden of caregivers and patients. But the diagnosis is often delayed, and such patients are complex because of their age, multiple pathologies, polypharmacy and the different stages of the disease. In addition, the symptoms of dementia can develop slowly or go unnoticed because such symptoms are similar to others caused by another more treatable and reversible diseases, so one should be alert for warning signs. Frequently, this lack of early diagnosis is because of the lack of knowledge about risk factors and issues related to the care or pharmacotherapy of the disease, what hinders diagnosis and care (Perry et al., 2008; Millard and Baune, 2009; Criddle, 2014).

Moreover, the pharmacological treatment must consider the different needs according to the stage of the disease. Cognitive deterioration gradually evolves from the first symptoms and may pre-exist for some time, producing a silent and presymptomatic neurodegeneration. Pharmacological therapy must recognize the symptomatic effect to preserve and improve cognition and a modifying effect to address the progression of the pathological process. Today, the only approved drugs are acetylcholinesterase inhibitors, which increase the acetylcholine levels in the cleft and non-competitive antagonists of glutamatergic receptors that regulate the activity of glutamate that is involved in learning and memory (Stella et al., 2015).

For all these reasons, the community pharmacist stands out for his privileged role as a health professional, for his knowledge and for being such an accessible professional for people. Pharmacists are those who must inform and advise on drugs, detect breaches, problems related to medicines and prevent diseases, offering pharmaceutical care that improves the quality of life of the patient. Such professionals can be trained to detect signs or characteristic symptoms of any pathology, performing tests and screenings to determine the possible referral to the practitioners (Climent et al., 2018). This turns pharmacies into excellent spaces to inform and disseminate best practices.

Thus, the aim of this research project is to describe and compare the level of AD knowledge among community pharmacists and general practitioners in Spain. The final objective is to find which are the most important gaps to develop the necessary programs that could help to solve them.

## Materials and Methods

### Bibliographic Revision

A bibliographic search was performed on Medline with AD and Alzheimer’s disease knowledge survey [Alzheimer’s disease knowledge survey (ADKS)] as keywords obtaining few results (**Table 1**) related to the knowledge of such issues among health professionals. Additionally, an article that measures the level of AD knowledge among caregivers and the general population was also reviewed to finally decide questions about methodological characteristics of the study (Jorge et al., 2018).

**Table 1:** Data of a bibliographic search of Alzheimer’s disease knowledge survey (ADKS) in health professionals to compare our results.

PLACE, YEAR, AUTHOR	*N*: PEOPLE RESPONDED TO THE SURVEY (% RESPONSE RATE)	METHODS	ADKS SCORE	HIGH KNOWLEDGE RELATED TO OWN DEMENTIA EXPERIENCE
Australia, 2013 Smyth W and cols	*N* = 360 participated (7.5%)	Online survey	Medicine: 26 Nursing: 23.9 Allied health: 23.87 Support staff: 22.14	Yes
Spain, 2019 Alacreu and cols	N = 578 (pharmacists) (8–42.3%) 104 (practitioners) (4.8–13%)	Web-based survey to pharmacist Manual distribution of hard copy of the survey to practitioners	Practitioners: 24.4 Pharmacists: 22.95	No
USA, 2015 Hughes ML and cols	*N* = 552 276 visit the web alz.org 276 control conditions % response rate unknown	Amazon’s Mechanical Turk Economical compensation for the participation	After visiting the Web site Health care workers: 25.13 No health care workers: 23.58 Control conditions: 22.32	Yes Brief exposure to the Web site about AD
Norway, 2012 Nordhus and cols	*N* = 956 psychologist of the Norwegian Psychological Association (NPA) (18%)	A web-based survey questionnaire	Psychologist: 24.10	Yes
Spain, 2018 C. Jorge	*N* = 419 215 caregivers 204 general population % response rate unknown	Hard copy of the survey at waiting room of the Cognitive Disorders Unit consultation	19.1 caregivers 18.8 general population	No
Malasia, 2017 Mat Nuri TH and cols	*N* = 445 (57.4%)	A web-based questionnaire, and manual distribution of hard copy of the survey (for those pharmacists who worked in nearby hospitals and health clinics	Health clinics pharmacists: 19.05 Hospital pharmacists: 18.47	-

### Participants and Study Description

A cross-sectional study was carried out between February and November 2018, implemented in two phases:

- **Phase I**: During this pilot phase, a survey was distributed to approximately 650 community pharmacists of the Spanish Society of Family and Community Pharmacy (SEFAC) of the Valencian Community. In this stage, answers from 274 pharmacists were obtained (with a participation of approximately 42.2%).
- **Phase II**: After the preliminary data of Phase I, the survey was extended with three questions (detailed in the following sections). In this stage, the target population was approximately 4,000 community pharmacists, associates of SEFAC in Spain, excluding the Valencian Community which had already participated in the previous phase, with a participation of 8%.

On the other hand, general practitioners of the Spanish Society of Primary Care Physicians (SEMERGEN), were also invited to participate (around a thousand general practitioners of this association) by means of an email in which the purpose of the study was detailed.

### Questionnaire and Data Collection

The survey spread among the participants consisted of anonymous demographic data of them plus the ADKS (Alzheimer’s disease knowledge survey) (**Table 2**) validated scale on the knowledge of Alzheimer’s disease (AD). ADKS is composed of a 30 items scale to investigate AD knowledge according to seven areas: impact on the disease (assessed by 3 items), risk factors (assessed by 6 items), course of the disease (assessed by 4 items), diagnosis (assessed by 4 items), care (assessed by 5 items), treatment (assessed by 4 items) and symptoms (assessed by 4 items) (Carpenter et al., 2009). Such scale was selected to measure the knowledge of AD for its demonstrated ease of use, reliability, and validity, and also for its suitability to be applied to different groups of respondents, including the general public, caregivers and health professionals (Smyth et al., 2013).

**Table 2:** ADKS questionnaire (Carpenter et al., 2009).

QUESTION NUMBER & ITEM	CORRECT ANSWER
1. People with Alzheimer’s disease are particularly prone to depression.	True
2. It has been scientifically proven that mental exercise can prevent a person from getting Alzheimer’s disease.	False
3. After symptoms of Alzheimer’s disease appear, the average life expectancy is 6 to 12 years.	True
4. When a person with Alzheimer’s disease becomes agitated, a medical examination might reveal other health problems that caused the agitation.	True
5. People with Alzheimer’s disease do best with simple, instructions giving one-step at a time.	True
6. When people with Alzheimer’s disease begin to have difficulty taking care of themselves, caregivers should take over right away.	False
7. If a person with Alzheimer’s disease becomes alert and agitated at night, a good strategy is to try to make sure that the person gets plenty of physical activity during the day.	True
8. In rare cases, people have recovered from Alzheimer’s disease.	False
9. People whose Alzheimer’s disease is not yet severe can benefit from psychotherapy for depression and anxiety.	True
10. If trouble with memory and confused thinking appears suddenly, it is likely due to Alzheimer’s disease.	False
11. Most people with Alzheimer’s disease live in nursing homes.	False
12. Poor nutrition can make the symptoms of Alzheimer’s disease worse.	True
13. People in their 30s can have Alzheimer’s disease.	True
14. A person with Alzheimer’s disease becomes increasingly likely to fall down as the disease gets worse.	True
15. When people with Alzheimer’s disease repeat the same question or story several times, it is helpful to remind them that they are repeating themselves.	False
16. Once people have Alzheimer’s disease, they are no longer capable of making informed decisions about their own care.	False
17. Eventually, a person with Alzheimer’s disease will need 24­hour supervision.	True
18. Having high cholesterol may increase a person’s risk of developing Alzheimer’s disease.	True
19. Tremor or shaking of the hands or arms is a common symptom inpeople with Alzheimer’s disease.	False
20. Symptoms of severe depression can be mistaken for symptoms of Alzheimer’s disease.	True
21. Alzheimer’s disease is one type of dementia.	True
22. Trouble handling money or paying bills is a common early symptom of Alzheimer’s disease.	True
23. One symptom that can occur with Alzheimer’s disease is believing that other people are stealing one’s things.	True
24. When a person has Alzheimer’s disease, using reminder notes is a crutch that can contribute to decline.	False
25. Prescription drugs that prevent Alzheimer’s disease are available.	False
26. Having high blood pressure may increase a person’s risk of developing Alzheimer’s disease.	True
27. Genes can only partially account for the development of Alzheimer’s disease.	True
28. It is safe for people with Alzheimer’s disease to drive, as long as they have a companion in the car at all times.	False
29. Alzheimer’s disease cannot be cured.	True
30. Most people with Alzheimer’s disease remember recent events better than things that happened in the past.	False

Moreover, it allows us to compare our results with other published research papers.

The demographic characteristics that were gathered were gender, age and years of professional practice. Only community pharmacists were asked about the type of pharmacy in which they develop their professional career according to inhabitants and about details of their role at the pharmacy (owner, responsible pharmacist or substitute). Moreover, after Phase I, it was decided to extend the survey by asking if the participants had close relatives with AD, to analyze their own dementia experience in relation to the disease.

Additionally, participants were also asked if they had received additional or specific training on AD, in order to assess the importance that this fact could have on the knowledge of the different areas of AD. Furthermore, the subjective knowledge the participant believes to have about AD on a scale of 0 (any knowledge) to 10 (well informed) was also asked. Nevertheless, this result was rescaled from 0 to 30 points as ADKS consists of a battery of 30 questions, whose possible answers are true or false, which assess the AD knowledge in seven areas.

Such sections are impacting on the disease, risk factors, course of the disease, diagnosis, care, treatment, and symptoms. Each area is evaluated by a different number of items, but not all of them are correlative. For example, the impact of the disease is assessed by items numbered as 1, 11 and 28. Each correct answer is scored with one point, thus the ADKS score ranges from 0 to 30 points. The estimated time to answer the questionnaire averaged 10 min.

### Statistical Analysis

Two surveys were created through Google forms for both community pharmacists (Ph) and general practitioners (Gp). The responses of each group were collected in two Excel sheets, version 2010. In the case of physicians, answers were obtained on paper and later stored by hand in the corresponding sheet. Once the storage process was finished, the data was cleaned in order to eliminate discrepancies, debugging the information of both groups in a common Excel sheet.

Subsequently, a statistical analysis was carried out using the R statistical software. After the descriptive analysis of the different variables, the association of the average ADKS score with the sociodemographic data was investigated.

Concretely, the *t*-test of independent samples was used in unilateral contrasts on the average ADKS score with respect to qualitative variables with two categories (gender, experience and training). To investigate possible significant differences between the average ADKS scores with respect to qualitative variables with more than two categories (age, work experience and type of pharmacist), ANOVA was used and, if their applicability hypotheses failed, Kruskal Wallis. Finally, to analyze the possible associations between the different items of the ADKS scale, the chi-square test and the exact Fisher test were used Statistical significance was assumed when the *p*-value <0.05 (*p* < 0.05).

## Results

Next, the most interesting conclusions obtained from studying the data are depicted. **Table 1** shows the results of a bibliographic search with the inclusion criterion of the ADKS survey to evaluate Alzheimer knowledge over health professionals. Among these studies, the people who responded to the survey varies between 360 and 956 individuals; in our study, 682 people responded. According to the way the survey was distributed (online, manual hard copy or with economical compensation) the rate of response varied between 7.5 and 57.4%.

In our study, obtained through an online survey, participation of 8% related to Spanish pharmacists, of which 42.3% pertained to the Valencian Community. But an inconvenience was the low participation of general practitioners in the online survey (only 4.8%), consequently, it forced a spreading of the responses of this group through the utilization of hard copies, making us of a conference held in SEMERGEN association to medical residents. 104 responses from general practitioners were obtained (38 *via* web and 66 printed questionnaires), increasing the participation to 13%.

At **Table 3**, it is possible to see a comparison between pharmacists and general practitioners. It summarizes sample size and ADKS average score, according to the demographic characteristics. As can be seen, surveys from 578 community pharmacists and 104 general practitioners were gathered. In both groups, women predominate (more than 60% of the participants). In summary, the ADKS average score in pharmacists was 22.95 ± 2.5 points compared to general practitioners which were 24.4 ± 2.3 points.

**Table 3:** Distribution of average ADKS scores according to different profiles.

	PHARMACISTS		GENERAL PRACTITIONERS	
	*N* (%)	ADKS score Mean ± SD	*N* (%)	ADKS score Mean ± SD
**GENDER** ** Men** ** Women**	140 (24.22%) 438 (75.78%)	22.8 ± 2.6 23.0 ± 2.4	41 (39.42%) 63 (60.58%)	24.6 ± 2.5 24.3 ± 2.2
**AGE** ** <30 years** ** 30–50 years** ** >50 years**	80 (13.84%) 315 (54.5%) 183 (31.66%)	22.6 ± 2.5 23.0 ± 2.5 23.0 ± 2.4	61 (58.65%) 24 (23.08%) 19 (18.27%)	24.9 ± 2.2 23.6 ± 2.6^b^ 23.8 ± 2.2
**WORK EXPERIENCE** ** <10 years** ** 10–20 years** ** >20 years**	186 (32.18%) 199 (34.43%) 193 (33.39%)	22.7 ± 2.4 23.2 ± 2.6 23.0 ± 2.5	77 (74.04%) 8 (7.69%) 19 (18.27%)	24.7 ± 2.3 24.8 ± 2.9 23.3 ± 1.9
**OWN DEMENTIA EXPERIENCE (ONLY PHASE II)** ** Yes** ** No**	178 (58.55%) 126 (41.45%)	22.8 ± 2.5 22.9 ± 2.5	51 (49.04%) 53 (50.96%)	24.5 ± 2.3 24.6 ± 2.5
**TRAINING (ONLY PHASE II)** ** Yes** ** No**	221 (72.7%) 83 (27.3%)	23.0 ± 2.5^a^ 22.3 ± 2.5	48 (46.15%) 56 (53.85%)	24.5 ± 2.3 24.3 ± 2.3
**TOTAL**	578 (100%)	22.95 ± 2.5	104 (100%)	24.4 ± 2.3

^a^p < 0.05 t test of independent samples, ^b^p < 0.05 ANOVA.

Additionally, it was discovered the ADKS average score is not influenced by gender, nor by work experience, nor by having a relative and/or close friend with AD. Regarding the question about whether respondents received specific training on AD, among the 302 pharmacists who answered in Phase II, 72.7% responded positively. Consequently, for this group, a significant difference in ADKS average score was observed in front of the pharmacists who had not received specific training. However, less than a half of the physicians received specific training on AD (46.15%) but this fact has no significant effect on the ADKS average score compared to those who had not received such training. Concerning age, it has an inverse association with ADKS in general practitioners, but not for pharmacists, as it can be seen in **Table 3**.

On the other hand, community pharmacists’ owners and adjuncts pharmacists obtained an average score significantly higher than the substitutes. And regarding the type of pharmacy, according to the number of inhabitants, where they develop their professional career, it seems to have no effect on their knowledge about the disease (see **Table 4**).

**Table 4:** Distribution of average ADKS scores according to different pharmacy types and pharmacists’ position.

	*N* (%)	ADKS score Mean ± SD
**TYPE OF PHARMACISTS** ** Owner** ** Responsible Pharmacist** ** Substitute Pharmacist**	316 (54.67%) 234 (40.48%) 28 (4.84%)	23.0 ± 2.5 23.1 ± 2.4 21.4 ± 3.0^a^
**TYPE OF PHARMACY** ** <2,500 people** ** <30,000 people** ** >30,000 people**	102 (17.65%) 154 (26.64%) 322 (55.71%)	22.9 ± 2.9 23.0 ± 2.4 22.9 ± 2.4
**TOTAL**	578 (100%)	22.95 ± 2.5

a p < 0.05 ANOVA.

**Figure 1:**
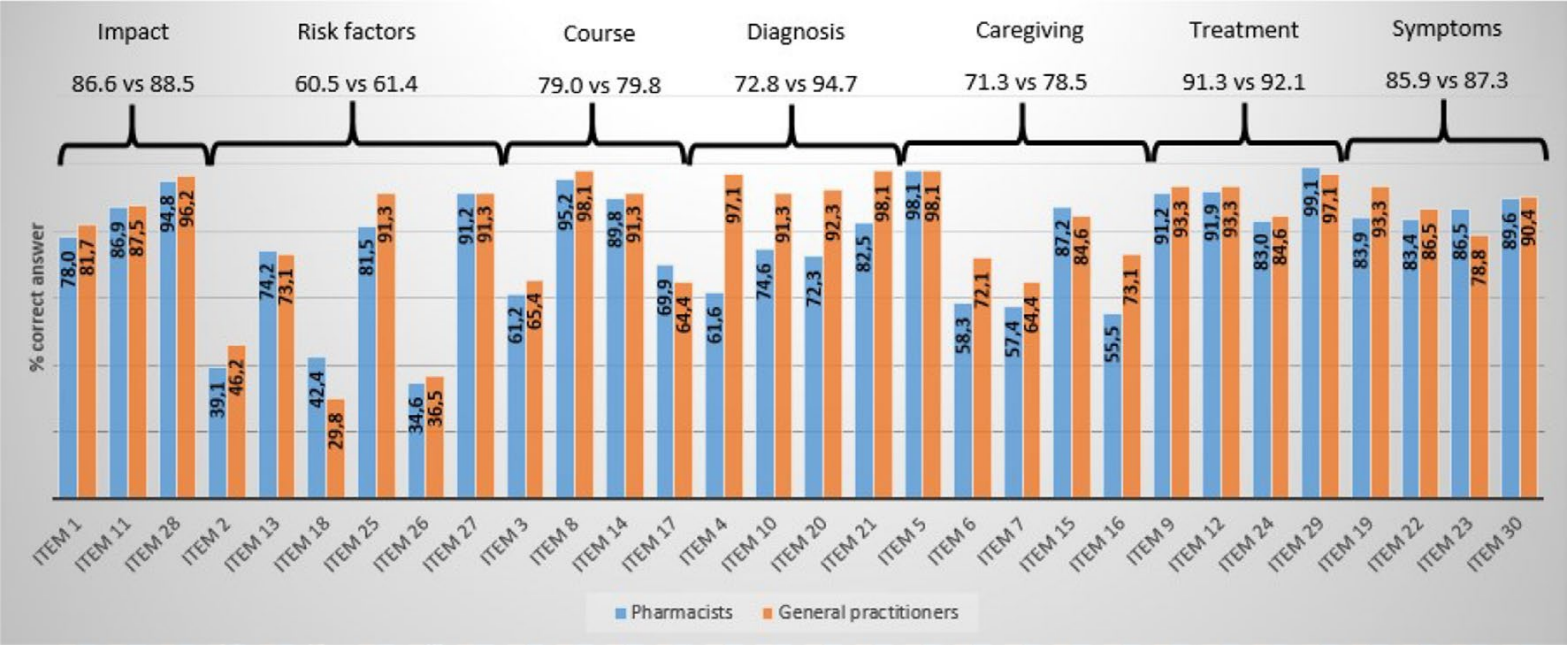
Comparison between pharmacists and general practitioners: percentage of correct answers per item and globally in each knowledge area.

**Figure 1** represents a comparison of the two participating groups (pharmacists and general practitioners). The percentage of correct answers within each item is summarized and the percentage of correct answers in each area is globally depicted, considering all the correct answers of each item that define the knowledge area.

### ADKS Knowledge Areas

Next, we slightly briefly describe the content of the ADKS areas and most relevant results. 

As it can be seen in **Figure 1** and **Table 5**, if we take into account the global knowledge both groups have about each area, pharmacists know from better to worse (percentage of correct answers): the treatment of the disease (91.3%), the impact on the disease (86.6%), the symptoms (85.9%), the course of the disease (79%), the diagnosis (72.8%), the care (71.3%) and finally, the risk factors (60.5%). Equivalently, general practitioners know from better to worse (percentage of correct answers): the diagnosis (94.7%), the treatment (92.1%), the impact on the disease (88.5%), the symptoms (87.3%), the course of the disease (79.8%), care (78.5%) and finally, risk factors (61.4%).

**Table 5:** Distribution of correct answers and average ADKS scores in seven knowledge areas related to impact, risk factors, course of disease, diagnosis, care giving, treatment and symptoms.

	PHARMACISTS		GENERAL PRACTITIONERS	
	% Correct (*N*)	ADKS score Mean ± SD	% Correct (*N*)	ADKS score Mean ± SD
**IMPACT (3 items)**	86.6 (1,501)	2.6 ± 0.6	88.5 (276)	2.7 ± 0.5
**RISK FACTORS (6 items)**	60.5 (2,098)	3.6 ± 1.2	61.4 (383)	3.7 ± 0.5
**COURSE OF DISEASE (4 items)**	79.0 (1,827)	3.2 ± 0.8	79.8 (332)	3.2 ± 0.8
**DIAGNOSIS (4 items)**	72.8 (1,682)	2.9 ± 0.9	94.7 (394)	3.8 ± 0.5
**CAREGIVING (5 items)**	71.3 (2,061)	3.6 ± 1	78.5 (408)	3.9 ± 1
**TREATMENT (4 items)**	91.3 (2,111)	3.7 ± 0.5	92.1 (383)	3.7 ± 0.6
**SYMPTOMS (4 items)**	85.9 (1,985)	3.4 ± 0.7	87.3 (363)	3.5 ± 0.6
**TOTAL (30 items)**	76.5 (17,340)	22.95 ± 2.5	81.4 (3,120)	24.4 ± 2.3

#### Impact of AD

AD knowledge about Impact is evaluated with items 1, 11 and 28 of the ADKS scale. This is the area evaluated with the least number of questions. In general, the tendency of successes in each item was similar for both groups, although general practitioners usually answer better. Item 1 states that those suffering from AD are particularly prone to depression. This true affirmation is the most unknown to the participants in this area (correct answers 78% Ph versus 81.7% Gp). Item 11 states that “*the majority of people suffering from AD live in nursing homes*.” Nevertheless, it is a false statement (correct answers 86.9% Ph versus 87.5% Gp), as it is the assertion of item 28: “*It is safe for people with AD to drive, as long as they have a companion in the car all the time*” (correct answers 94.8% Ph versus 96.2% Gp). This is the best-known item for both groups in this area of knowledge.

#### Risk Factors for AD

The most representative area in the ADKS scale is the area related to knowledge about risk factors, since such area is evaluated through 6 items (2, 13, 18, 25, 26, and 27): it seems that both groups have difficulties in determining the affirmation of item 2 (correct answers 39.1% Ph versus 46.2% Gp): “*It has been scientifically proven that mental exercise can prevent a person from getting AD*,” which is false. And, the item 13: “*People in their 30s can have AD*,” what is a true statement, well known by pharmacists (correct answers 74.2% Ph versus 73.1% Gp).

The ignorance of both groups, especially of general practitioners, is striking about the affirmation of item 18 (correct answers 42.4% Ph versus 29.8% Gp): “*Having high cholesterol may increase a person’s risk of developing AD*,” which is a true statement. Moreover, item 25 is a false affirmation that both groups can identify without too much difficulty (correct answers 81.5% Ph versus 91.3% Gp): “*Prescription drugs that prevent AD are available*.” The affirmation of item 26: “*Having high blood pressure may increase a person’s risk of developing AD*,” which is a true affirmation which both groups struggle to identify (correct answers 34.6% Ph versus 36.5% Gp). Finally, item 27: *“Genes can only partially account for the development of AD*,” it is also a true statement known by the majority (correct answers 91.2% Ph versus 91.3% Gp).

#### The Course of AD

The course of the disease is a knowledge area evaluated by 4 items (3, 8, 14 and 17). The statement of item 3: “*After the symptoms of AD appear, the average life expectancy is between 6 to 12 years*,” although it is a correct statement, it seems to be the most unknown aspect for the participants in this area (correct answers 61.2% Ph versus 65.4% Gp). Nevertheless, no problems have been detected to identify that the affirmation of item 8: “*In rare cases, people have recovered from AD*,” is false (correct answers 95.2% Ph versus 98.1% Gp). Moreover, regarding the item 14: “*A person with AD becomes increasingly likely to fall down as the disease gets worse*,” it has been identified as true without much difficulty (correct answers 89.8% Ph versus 91.3% Gp). Finally, there was a problem in identifying whether the assertion of item 17 is true: “*Eventually, a person with AD will need 24-hour supervision*” (correct answers 69.9% Ph versus 64.4% Gp).

#### Diagnosis of AD

The next area evaluated is about knowledge of the disease diagnosis. The ADKS scale dedicates 4 items (4, 10, 20 and 21) for such issue. This aspect is the one that stands out because of the superior knowledge of the general practitioners against pharmacists. The affirmation of item 4: “*When a person with AD becomes agitated, a medical examination might reveal alternative health problems that caused the agitation*,” although it is true, among pharmacists it is the most unknown statement. On the contrary, general practitioners knew very well such issue (correct answers 61.6% Ph versus 97.1% Gp). Furthermore, within the ADKS scale there is an item with the greatest differences between pharmacists and general practitioners regarding the item 10: “*If troubles with memory and confused thinking appear suddenly, it is likely due to AD*,” which is false (correct answers 74.6% Ph versus 91.3% Gp). Finally, in the following two true statements, item 20: “*Symptoms of severe depression can be mistaken with AD symptoms*” (correct answers 72.3% Ph versus 92.3% Gp) and item 21: “*AD is a type of dementia*,” a greater knowledge in general practitioners than pharmacists have been detected (correct answers 82.5% Ph versus 98.1% Gp).

#### AD Care

The area of care is evaluated through 5 items. The affirmation of item 5: “*People with AD do best with simple instructions, given one step at a time*,” is true and also well known by both groups (Both 98.1%). The affirmation of item 6: “*When people with AD begin to have difficulty taking care of themselves, caregivers should take over right away*,” is false (correct answers 58.3% Ph versus 72.1% Gp). Moreover, the affirmation of item 7: “*If a person with AD becomes alert and restless at night, a good strategy is to try to make sure the person gets plenty of physical activity during the day*,” is correct but it is one of the most unknown issues for both collectives within the AD care area (correct answers 57.4% Ph versus 64.4% Gp). Additionally, the following two statements are false. Item 15: “*When people with AD repeat the same question or story several times, it is helpful to remind them they are repeating themselves*,” it is one of the 5 items better known by pharmacists than general practitioners (correct answers 87.2% Ph versus 84.6% Gp) and item 16: “*Once people present with AD, they are no longer capable of making informed decisions about their own care*,” a higher percentage of knowledge was obtained by general practitioners (correct answers 55.5% Ph versus 73.1% Gp).

#### Treatment of AD

The treatment area is collected by four questions. In this area, both groups answer almost all questions correctly, without any significant difference, as it can be seen in **Figure 1**. It is the area with the highest scores of the entire ADKS scale, exceeding 90% of correct answers. Related to item 9: “*People whose AD is not yet severe can benefit from psychotherapy for depression and anxiety*,” it is a true affirmation but not everybody answered correctly (correct answers 91.2% Ph versus 93.3% Gp). And with respect to item 12: “*Poor nutrition can make the symptoms of AD getting worse*”; it is also a true statement (correct answers 91.9% Ph versus 93.3% Gp). Besides item 24: “*When a person has AD, using reminder notes is a crutch that can contribute to declining*,” is a false statement. The most unknown within the area (correct answers 83% Ph versus 84.6% Gp) and finally, in relation to the item 29: “*AD cannot be cured*,” is a true statement, and the percentage of knowledge in this item is higher in pharmacists (correct answers 99.1% Ph versus 97.1% Gp).

#### Symptoms of AD

At last, there are 4 items dedicated to assessing disease symptoms knowledge. In general, the percentage of correct answers is reasonably good for both groups, as it can be seen in **Figure 1**. It is the third area with the best scores, relating to the treatment and impact of the disease, respectively. Item 19: “*Tremor or shaking of the hands or arms is a common symptom in people with AD*,” is a false claim (correct answers 83.9% Ph versus 93.3% Gp). With respect to item 22: “*Trouble, when handling money or paying bills, is a common early symptom of AD*,” it is a true affirmation but once again not everybody answered correctly (correct answers 83.4% Ph versus 86.5% Gp). Item 23: “*One symptom that can occur with AD is believing that other people are stealing one’s things*,” is a true statement and pharmacists know better than general practitioners (correct answers 86.5% Ph versus 78.8% Gp). In the end, item 30: “*Most people with AD remember better recent events than things happened in the past*,” is a false claim (correct answers 89.6% Ph versus 90.4% Gp).

In both **Figure 1** and **Table 5**, the comparison between pharmacists and general practitioners appears on the overall percentage of successful answers within each area. In **Table 5**, this percentage is accompanied by the number of successful answers and the ADKS average score within each area. Moreover, to mention the maximum score that can be obtained in each area corresponds to the number of items that define it. The lowest average score per area, in both groups, is obtained in the area related to risk factors. The overall ADKS average score is higher in general practitioners and is motivated by their superior knowledge in the area of diagnosis.

Additionally, the association of sociodemographic data on average scores in each knowledge area was also analyzed among pharmacists (**Table 6**) and general practitioners (**Table 7**). Although gender, age, work experience and having a family member or close friendship with AD has no significant association with the average ADKS score, there are other significant associations within specific areas that have been observed. For example, male pharmacists’ score significantly less in the area of risk factors and higher in the diagnosis than women. Age has an inverse association with the average score in care but directs with symptoms average score. Moreover, work experience reproduces practically the same behavior as the age variable. And pharmacists who have a family member or close friendship with AD, score more significantly in symptoms. Finally, having specific training on the disease implies a higher ADKS average score, motivated by a higher average score in diagnosis.

**Table 6:** ADKS average score in each knowledge area, for pharmacists.

	N (%)	ADKS score (Mean ± SD)	KNOWLEDGE AREAS FOR PHARMACISTS						
			Impact (3 items) (Mean ± SD)	Risk Factors (6 items) (Mean ± SD)	Course (4 items) (Mean ± SD)	Diag. (4 items) (Mean ± SD)	Caregiv. (5 items) (Mean ± SD)	Treat. (4 items) (Mean ± SD)	Sympt. (4 items) (Mean ± SD)
**SEX** **Men** **Women**	140 (24.22%) 438 (75.78%)	22.8 ± 2.6 23.0 ± 2.4	2.6 ± 0.6 2.6 ± 0.6	3.5 ± 1.1^a^ 3.7 ± 1.2	3.1 ± 0.8 3.2 ± 0.8	3.0 ± 0.9^a^ 2.9 ± 0.9	3.6 ± 1.0 3.6 ± 1.0	3.6 ± 0.6 3.7 ± 0.5	3.4 ± 0.7 3.4 ± 0.7
**AGE** **<30 years** **30–50 years** **>50 years**	80 (13.84%) 315 (54.5%) 183 (31.66%)	22.6 ± 2.5 23.0 ± 2.5 23.0 ± 2.4	2.5 ± 0.6 2.6 ± 0.6 2.6 ± 0.6	3.7 ± 1.1 3.6 ± 1.2 3.6 ± 1.2	3.0 ± 0.8 3.1 ± 0.8 3.3 ± 0.8	2.7 ± 0.9 2.9 ± 0.9 3.0 ± 0.9	3.7 ± 1.0 3.7 ± 1.0 3.3 ± 1.0^b^	3.6 ± 0.6 3.7 ± 0.5 3.7 ± 0.6	3.2 ± 0.8 3.4 ± 0.7 3.6 ± 0.6^c^
**WORK EXPERIENCE** **<10 years** **10–20 years** **>20 years**	186 (32.18%) 199 (34.43%) 193 (33.39%)	22.7 ± 2.4 23.2 ± 2.6 23.0 ± 2.5	2.6 ± 0.6 2.6 ± 0.6 2.6 ± 0.6	3.6 ± 1.1 3.7 ± 1.2 3.6 ± 1.2	3.1 ± 0.8 3.1 ± 0.8 3.3 ± 0.8	2.8 ± 0.8 3.0 ± 0.9 2.9 ± 0.9	3.7 ± 1.0 3.7 ± 0.9 3.3 ± 1.0^b^	3.6 ± 0.6 3.7 ± 0.5 3.7 ± 0.5	3.3 ± 0.8^c^ 3.4 ± 0.7 3.6 ± 0.7
**BASIC KNOWLEDGE (Phase II)** ** Yes** ** No**	178 (58.55%) 126 (41.45%)	22.8 ± 2.5 22.9 ± 2.5	2.6 ± 0.6 2.7 ± 0.5	3.5 ± 1.2 3.7 ± 1.1	3.1 ± 0.8 3.1 ± 0.8	2.9 ± 0.9 2.9 ± 0.9	3.5 ± 1.0 3.6 ± 1.0	3.6 ± 0.6 3.6 ± 0.6	3.5 ± 0.7^a^ 3.4 ± 0.7
**TRAINING (Phase II)** ** Yes** ** No**	221 (72.7%) 83 (27.3%)	23.0 ± 2.5^a^ 22.3 ± 2.5	2.6 ± 0.6 2.6 ± 0.6	3.6 ± 1.2 3.5 ± 1.2	3.1 ± 0.8 3.1 ± 0.8	2.9 ± 0.9^a^ 2.7 ± 1	3.6 ± 1.0 3.6 ± 0.9	3.7 ± 0.5 3.6 ± 0.6	3.5 ± 0.7 3.3 ± 0.8
**TOTAL**	578 (100%)	22.95 ± 2.5	2.6 ± 0.6	3.6 ± 1.2	3.2 ± 0.8	2.9 ± 0.9	3.6 ± 1	3.7 ± 0.5	3.4 ± 0.7

^a^p < 0.05 t test of independent samples, ^b^p < 0.05 ANOVA, ^c:^p < 0.05 Kruskal Wallis.

**Table 7:** ADKS average score in each knowledge area, for general practitioners.

	*N* (%)	ADKS score (Mean± SD)	AREAS OF KNOWLEDGE FOR GENERAL PRACTITIONERS						
			Impact (3 items) (Mean ± SD)	Risk Factors (6 items) (Mean ± SD)	Course (4 items) (Mean ± SD)	Diag. (4 items) (Mean ± SD)	Caregiv. (5 items) (Mean ± SD)	Treat. (4 items) (Mean ± SD)	Sympt. (4 items) (Mean ± SD)
**SEX** ** Men** ** Women**	41 (39.42%) 63 (60.58%)	24.6 ± 2.5 24.3 ± 2.2	2.8 ± 0.5 2.6 ± 0.6	4.0 ± 1.2^a^ 3.5 ± 1.1	3.2 ± 0.8 3.2 ± 0.8	3.8 ± 0.5 2.8 ± 0.5	3.9 ± 1.0 3.9 ± 1.0	3.7 ± 0.6 3.7 ± 0.6	3.3 ± 0.7^a^ 3.6 ± 0.5
**AGE** ** <30 years** ** 30–50 years** ** >50 years**	61 (58.65%) 24 (23.08%) 19 (18.27%)	24.9 ± 2.2 23.6 ± 2.6^b^ 23.8 ± 2.2	2.7 ± 0.5 2.5 ± 0.7 2.8 ± 0.4	3.8 ± 1.1 3.5 ± 1.3 3.6 ± 1.1	3.2 ± 0.8 3.2 ± 0.8 3.2 ± 0.7	3.9 ± 0.3^c^ 3.7 ± 0.5 3.5 ± 0.6	4.2 ± 0.9^b^ 3.6 ± 1.1 3.5 ± 1.2	3.7 ± 0.5 3.6 ± 0.7 3.6 ± 0.7	3.5 ± 0.7 3.5 ± 0.5 3.6 ± 0.6
**WORK EXPERIENCE** ** <10 years** ** 10–20 years** ** >20 years**	77 (74.04%) 8 (7.69%) 19 (18.27%)	24.7 ± 2.3 24.8 ± 2.9 23.3 ± 1.9	2.6 ± 0.6 3.0 ± 0.0 2.7 ± 0.5	3.8 ± 1.2 3.6 ± 1.1 3.4 ± 1.1	3.2 ± 0.8 3.6 ± 0.5 3.0 ± 0.6	3.9 ± 0.3 3.8 ± 0.5 3.4 ± 0.6^c^	4.1 ± 0.9^b^ 3.2 ± 1.6 3.6 ± 1.1	3.7 ± 0.6 3.9 ± 0.4 3.6 ± 0.8	3.5 ± 0.6 3.6 ± 0.5 3.5 ± 0.6
**BASIC KNOWLEDGE** ** Yes** ** No**	51 (49.04%) 53 (50.96%)	24.5 ± 2.3 24.6 ± 2.5	2.6 ± 0.6 2.7 ± 0.5	3.6 ± 1.2 3.8 ± 1.1	3.1 ± 0.7 3.3 ± 0.8	3.7 ± 0.5 3.8 ± 0.4	3.9 ± 1.0 3.9 ± 1.0	3.7 ± 0.6 3.7 ± 0.6	3.6 ± 0.6 3.4 ± 0.6
**TRAINING** ** Yes** ** No**	48 (46.15%) 56 (53.85%)	24.5 ± 2.3 24.3 ± 2.3	2.7 ± 0.5^a^ 2.5 ± 0.6	3.7 ± 1.1 3.7 ± 1.3	3.2 ± 0.7 3.2 ± 0.9	3.7 ± 0.5 3.9 ± 0.4	4.0 ± 1.0 3.8 ± 1.1	3.7 ± 0.6 3.7 ± 0.7	3.5 ± 0.6 3.5 ± 0.7
TOTAL	104 (100%)	24.4 ± 2.3	2.7 ± 0.5	3.7 ± 0.5	3.2 ± 0.8	3.8 ± 0.5	3.9 ± 1	3.7 ± 0.6	3.5 ± 0.6

^a^p < 0.05 t test of independent samples, ^b^p < 0.05 ANOVA, ^c^p < 0.05 Kruskal Wallis.

To summarize, the behavior of general practitioners’ changes with respect to pharmacists. Men score significantly more on risk factors, but significantly less on symptoms than women. The ADKS average score is significantly higher in general physicians younger than 30 years old, motivated by greater knowledge in the diagnosis and care areas. In terms of work experience, general practitioners score significantly better in diagnosis and care. Nevertheless, the ADKS average score is not affected by work experience. Having a relative or close friendship with the disease has no effect on the average scores in any AD knowledge areas. Additionally, having received specific training on AD provides the corresponding significant higher mean score in the impact area.

In the end, substitute pharmacists score significantly less in treatment with respect to adjuncts and owners. Furthermore, adjunct pharmacists score significantly higher on care and lower on symptoms than others. Finally, the type of pharmacy in which the pharmacist exercises, according to the number of inhabitants, has no effect on the average score in any AD knowledge areas (**Table 8**).

**Table 8:** ADKS average score in the different knowledge area according to the pharmacists profile.

	N (%)	ADKS score (Mean ± SD)	AREAS OF KNOWLEDGE FOR PHARMACISTS						
			Impact (3 items) (Mean ± SD)	Risk Factors (6 items) (Mean ± SD)	Course (4 items) (Mean ± SD)	Diag. (4 items) (Mean ± SD)	Caregiv. (5 items) (Mean ± SD)	Treat. (4 items) (Mean ± SD)	Sympt. (4 items) (Mean ± SD)
**TYPE OF PHARMACISTS** **Owner** **Responsible Pharmacist** **Pharmacist Substitute**	316 (54.67%) 234 (40.48%) 28 (4.84%)	23.0 ± 2.5 23.1 ± 2.4 21.4 ± 3.0^a^	2.6 ± 0.6 2.6 ± 0.6 2.4 ± 0.7	3.6 ± 1.2 3.7 ± 1.2 3.5 ± 1.4	3.2 ± 0.8 3.1 ± 0.8 3.1 ± 0.7	2.9 ± 0.9 2.9 ± 0.9 2.6 ± 0.9	3.5 ± 0.9 3.7 ± 1.0^a^ 3.2 ± 0.9	3.7 ± 0.5 3.7 ± 0.5 3.3 ± 0.7 ^a^	3.5 ± 0.7 3.3 ± 0.8^b^ 3.4 ± 0.7
**TYPE OF PHARMACY** **<2,500 people** **<30,000 people** **>30,000 people**	102 (17.65%) 154 (26.64%) 322 (55.71%)	22.9 ± 2.9 23.0 ± 2.4 22.9 ± 2.4	2.5 ± 0.7 2.6 ± 0.6 2.6 ± 0.6	3.5 ± 1.3 3.7 ± 1.2 3.6 ± 1.2	3.2 ± 0.8 3.2 ± 0.7 3.1 ± 0.8	3.0 ± 0.9 2.9 ± 0.9 2.9 ± 0.9	3.5 ± 1.0 3.5 ± 1.0 3.6 ± 1.0	3.7 ± 0.5 3.7 ± 0.6 3.6 ± 0.5	3.5 ± 0.7 3.4 ± 0.7 3.4 ± 0.8
**TOTAL**	578 (100%)	22.95 ± 2.5	2.6 ± 0.6	3.6 ± 1.2	3.2 ± 0.8	2.9 ± 0.9	3.6 ± 1	3.7 ± 0.5	3.4 ± 0.7

ADKS average score in the different knowledge area according to different pharmacy types and pharmacists’ position. ^a^p < 0.05 ANOVA, ^b^p < 0.05 Kruskal Wallis.

### Associations Between Items

**Figure 2:**
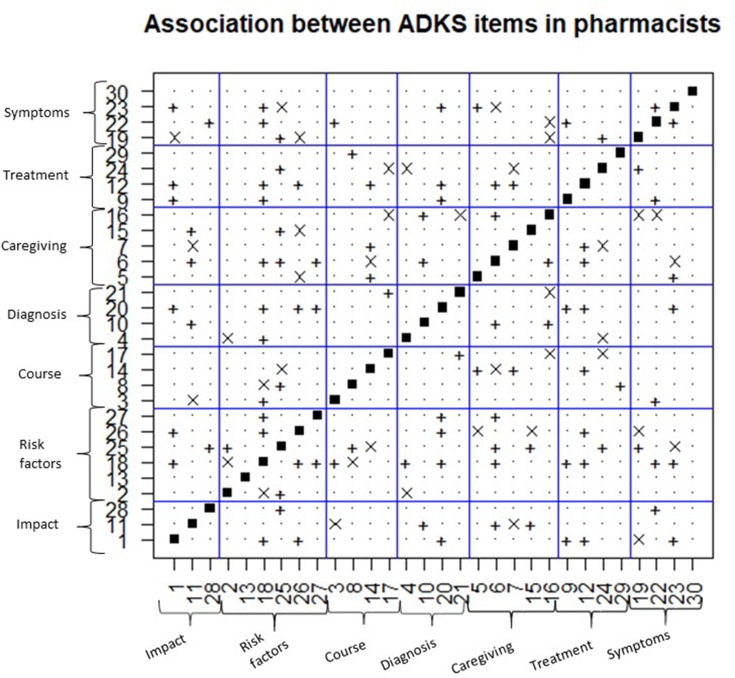
Associations between the items of the ADKS scale in pharmacists. +, indicates positive association between the items; x, indicates negative association between the items. *p* < 0.05 chi-square test.

**Figure 3:**
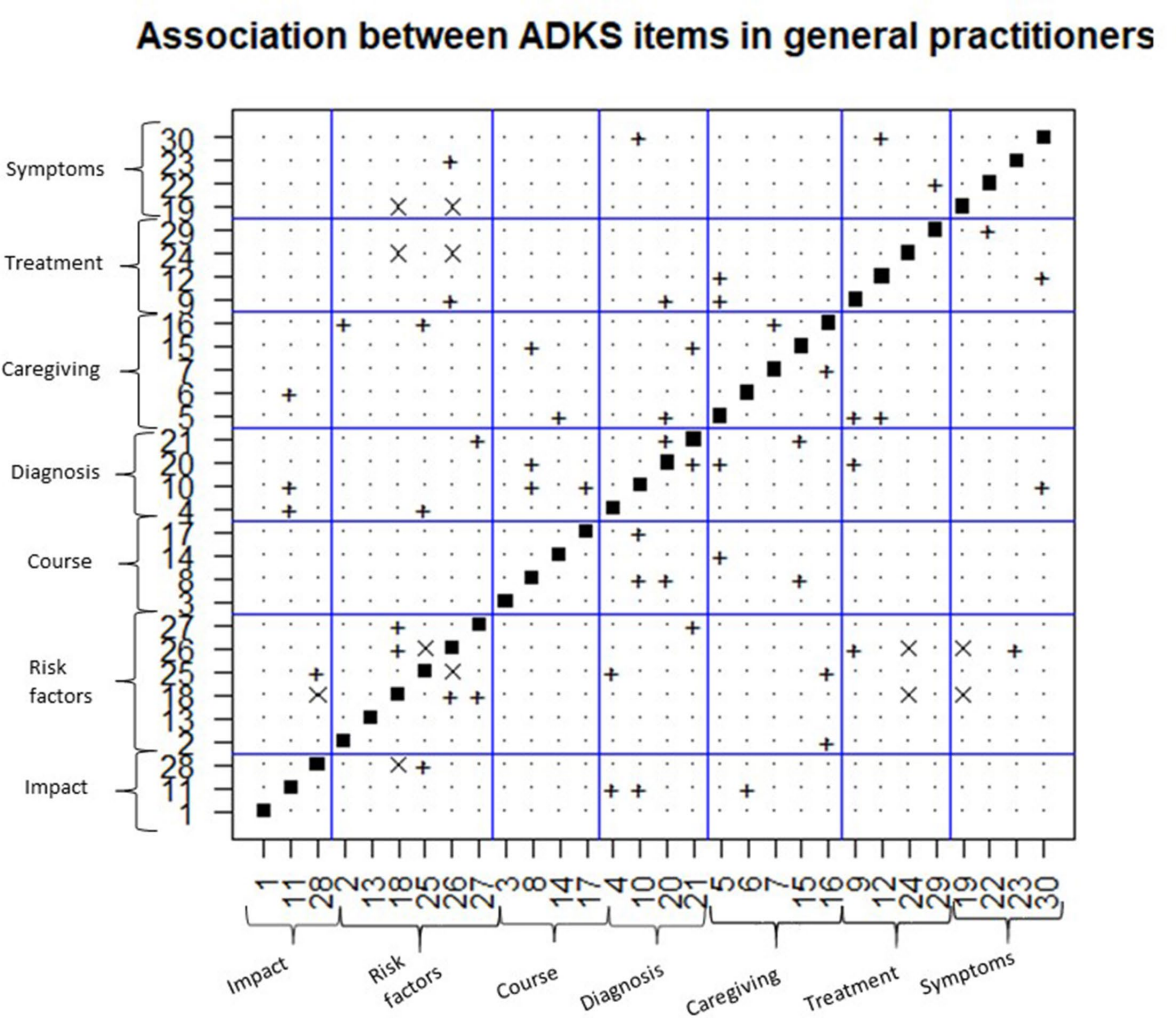
Associations between the items of the ADKS scale in general practitioners. +, indicates positive association between the items; x, indicates negative association between the items. *p* < 0.05 chi-square test.

The association between the responses of the ADKS items has also been analysed for both groups. The result obtained has been organized in **Figure 2** (for pharmacists) and **Figure 3** (for general practitioners). The abscissa and the ordinate axis contain the items of the ADKS scale, and they are grouped by seven knowledge areas. Each figure is symmetric with respect to the main diagonal because when a significant association between item *i* and *j* is determined, there is also the same association between item *j* and *i*, according to the chi-square test.

The main diagonal is discarded from the analysis, as it has no statistical interest. The “*+*” symbol indicates that there is a significant positive association between items *i* and *j*. That is, in general when the item *i* is correctly answered, the tendency is to answer item *j* also correctly and vice-versa. On the contrary, the symbol “x” indicates the association between items is negative. That is, when the item *i* is correctly answered, there is a tendency to answer item *j* incorrectly and vice versa.

According to **Figure 2** (pharmacists), amongst 435 associations evaluated between the different pairs of different items, there are 69 significant (49 positive and 20 negative). Independence is assumed in the response between the items of the same area, in four of the seven knowledge areas: impact on the disease, course of the disease, diagnosis, and treatment of the disease. That is, knowing the answer in any of the different items in these areas does not imply that you will also know the correct answer of other items of the same ​​knowledge area.

There are four associations between the items corresponding to the knowledge about risk factors, an association between the items in the care area and an association between items in the symptoms area. Item 13 and item 30 are the only two items on the ADKS scale whose response is not associated with the response of the rest of the items for pharmacists. Item 18 is the one with the highest number of associations with the rest: (true affirmation) is a question about risk factors whose response is associated with 13 items distributed by different knowledge areas.

On the other hand, **Figure 3** is devoted to the results obtained with general practitioners. Among the 435 associations evaluated, 33 were significant (27 positives and 6 negatives). This time, independence is also assumed between responses of items of the same area, in four of the seven knowledge areas: impact on the disease, course of the disease, treatment and symptoms. There are three associations between items in the risk factors area, an association between items in the diagnosis area of the disease and an association between items in the care area. In this case, item 1, 13 and 3 are not associated with the rest of the items.

There are seven coincidences of association between both groups: item 11, it is positively associated with item 10 and with item 6. Item 28 is positively associated with item 25, and item 18 is associated with item 26 and item 27. With respect to item 14, it is associated with item 5. Nevertheless, item 20 is associated with item 12 (**Figure 3**).

## Discussion

This is the first study in Spain that tackles the level of AD knowledge among pharmacists and practitioners using the ADKS survey. Nevertheless, there is only an article that measures the level of AD knowledge among caregivers and general population that has just been published and allows us to have an overall comparison in order to describe the view of AD knowledge in Spain, both among health professionals (practitioners scored 24.4 and pharmacists scored 22.95), caregivers (19.2) and general population (18.9) (Jorge et al., 2018).

### Participation

The response rate among participants was variable. Spanish pharmacists responded to the survey about 8%; similar to the data obtained among the health staff in Australia (Smyth et al., 2013). Nonetheless, this rate increases to 42.3%, if we consider only the Valencian Community region. Such figures may be because of a study on screening for cognitive impairment that is now taking place in our university and has been published on numerous occasions in the local press, which makes pharmacists more aware of the participation (Climent et al., 2018). Nevertheless, the practitioner’s response *via* the Internet survey was very low (4.8%). We increased this result through a manual distribution of hard copies survey during a medical meeting, obtaining a 13% of total participation, this mixed methodology is used in other papers (Mat Nuri et al., 2017).

### Score

The score attained by the Spanish pharmacists (22.95), though better than the Malaysian pharmacists (19.05) (Mat Nuri et al., 2017), it was lower than scores obtained by Norwegian psychologists (24.10) and Australian health professionals (23.9) (Smyth et al., 2013). However, the score obtained by the Spanish practitioners was 24.4, similar to the Norwegian psychologists and higher than Australian practitioners (Smyth et al., 2013). Thus, although Spanish pharmacists and practitioners got a higher score and personal knowledge of AD, it was not associated with greater awareness.

Pharmacist and practitioner participants scored at least 60% in all categories and above 80% at: diagnostic, treatment and symptoms. The category of diagnosis received the highest mean percentage of correct responses (94.7%) among practitioners and the category of treatment obtained the highest with pharmacists (91.3%). However, interesting issues that have been observed are that a lower knowledge (60% of correct answers) was found to those related to risk factors. The ignorance about the hypercholesterolemia or hypertension as risk factors for the disease was striking. In fact, from the association tables, it is possible to observe that items 18 and 26 are associated in both groups in a positive way, that is, when one is incorrectly answered the other one is also incorrect and vice versa. The knowledge of both items is positively associated; thus, it should be advisable to educate both groups (and in general the health personnel) that cardiovascular risk factors have a direct impact on the AD. Anyway, it is remarkable that knowledge of modifiable risk factors for dementia seems low in most studies (Hudson et al., 2012; Nordhus et al., 2012; Smyth et al., 2013; Mat Nuri et al., 2017; Jorge et al., 2018).

There are many pieces of evidence that suggest a strong correlation between cardiovascular disease in middle age and dementia. Ideal cardiovascular health in the adult stage and its maintenance in middle age were related as an improvement in psychomotor speed, executive function and verbal memory. The main factors associated with high cardiovascular risk in middle age are hypertension, heart ejection fraction, obesity, diabetes, and dyslipidemia (Reis et al., 2013; Yaffe et al., 2014; Nepal et al., 2017).

Moreover, a lack of knowledge was also observed in aspects related to the care of patients and the course of the illness, such as the ADKS questions as “*if the patient wakes up at night a good strategy is to keep physical activity during the day*” or “*if there is a need for taking charge of the patient immediately when he/she starts to have self-care problems*.”

Finally, patient care demands a comprehensive education. Programs, with information about the degree of severity of dementia, would provide participants with specific skills to allow a good screening for cognitive decline. A possible area in which more research is needed is to examine what type of cognitive stimulation is associated with better results in decelerating the AD evolution of the patients.

### Expanding the Role of the Pharmacist

Cardiovascular risk factors (diabetes, obesity, smoking, hypertension and high total cholesterol level) are treatable. Management of these risk factors should lead to a reduction in the incidence of cognitive impairment (Steven, 2015; Santos et al., 2017). High cardiovascular risk factors at mid-life may increase the risk of dementia and AD later in life, each of them increasing the risk around two times (Kivipelto et al., 2005).

The effectiveness of community pharmacy-based public health interventions in reducing these risk factors and preventive activities in chronic illness as hypertension and hypercholesterolemia were shown. These interventions include health education, medication review, blood pressure measurement and adherence to treatment. The effect of pharmacist care on patients with cardiovascular diseases has been associated with an enhanced adherence to treatment, significant reduction in blood pressure and serum cholesterol (Santschi et al., 2011).

It has been shown that pharmacists are already acting to control cardiovascular risk factors, but a wider knowledge of the relationship of these factors with AD is needed in order to have more reasons to persuade patients to modify such silent risk factors.

One of the most common mistakes found in many studies is that dementia is a normal phase of aging. Moreover, there is a lack of knowledge about the point at which problems memory loss related to age become enough serious to indicate a high probability of dementia. This finding is not surprising given that many health professionals, even general practitioners trained to diagnose and treat diseases like dementia, often experience similar concerns (Vernooij‐Dassen et al., 2005; Cahill et al., 2008).

Early screening of dementia is the precondition for improving dementia care, but diagnosis often occurs at the end of the disease process. Thus, evaluation tools such as the ADKS can play an important role in identifying educational deficiencies to facilitate better care and treatment of people with dementia. Moreover, it can highlight which specific areas of dementia knowledge are lacking. In that case, knowledge about risk factors is important from the perspective of the general population health and risk profiles.

### Interprofessional Collaboration of General Practitioners and Community Pharmacists

There is evidence for the positive effect of community pharmacists on health care about an interprofessional collaboration of the latter and general practitioners. However, pharmacists remain an underutilized resource in primary health care (Löffler et al., 2017).

Additionally, community pharmacists are very participative professionals and already play a crucial role in preventing cardiovascular risk factors, such as hypertension and hypercholesterolemia, both also risks factors of dementia, but not all of them know the relationship to AD.

Pharmacists are accessible, reliable and respected health professionals. Greater participation of the pharmacist in the care of people with AD could improve clinical results and quality of life of patients and caregivers. Thus, innovative approaches should be developed to expand the pharmacist’s participation in AD (Skelton, 2008).

#### Limitations of the Cross-sectional Design of the Study

The main problem encountered was the low participation of general practitioners in the online survey, that forced us to spread out the responses of this group through the utilization of hard copies, trying to increase participation in this way. The sample used to carry out the inference study comes from two health professional societies (SEFAC and SEMERGEN), whose associates should meet the requirement of having a corresponding university degree. Therefore, the participants of the anonymous survey objectively represent the profile of both professionals: pharmacists and general practitioners. It minimizes the inclusion bias of such professional collectives. Moreover, the comparison of the average level of knowledge about Alzheimer’s disease has been objectively carried out using an internationally validated index, the ADKS survey, which minimizes a possible information bias.

## Conclusions

Spanish health professionals’ knowledge about AD seems to be high based on the results we have obtained in this study. Nevertheless, some issues are remarkable, e.g. when health professionals were interviewed, it was not well known that the influence of high blood pressure or high levels of blood lipids at an early stage of life, may have an important role in the development of AD in old age. This fact demonstrates there is a need to develop and implement new training strategies to improve such knowledge to the medical and pharmaceutical professionals in the field of AD risk factors. Patients value the services received from community pharmacists therefore the offer of novel services into new areas as dementias could be an opportunity.

Moreover, it is important to highlight the importance of additional training in AD risk factors in the clinical setting, in order to be able to carry out preventive and early detection activities. The better educated the health staff in general is and the greater knowledge it gathers of risk factors and of global prevention, the greater the benefit for patients will be.

A multidisciplinary collaborative approach between the pharmacist and the referring physician within a patient-centered model of care has proven to be particularly effective in improving the control of risk factors and the promotion of patients’ health.

## Data Availability

The raw data supporting the conclusions of this manuscript will be made available by the authors, without undue reservation, to any qualified researcher.

## Ethics Statement

The participation was anonymous. Before completing the initial scale, the volunteer respondents were assured of confidentiality and informed that sending a completed online survey was considered consent for the inclusion of data not identified in the reports. This study was reviewed and approved by the Research Ethics Committee of CEU Cardenal Herrera University (approval No. CEI18 / 27) in February 2018.

## Author Contributions

MaA contributed to the data collection. JP and MóA assisted in data analysis, interpretation, and write-up. MC and VG assisted in data collection and write-up. LM assisted in concept, design, critical revision, and write-up. All authors listed have made a substantial, and direct contribution to the work, and approved the final manuscript as submitted.

## Funding

Know Alzheimer Foundation and Universidad CEU Cardenal Herrera have funded part of the research.

## Conflict of Interest Statement

The authors declare that the research was conducted in the absence of any commercial or financial relationships that could be construed as a potential conflict of interest.

## References

[B1] Cahill S. Clark M. O’Connell H. Lawlor B. Coen R. F. Walsh C. (2008). The attitudes and practices of general practitioners regarding dementia diagnosis in Ireland. Int. J. Geriatr. Psychiatry 23, 663–669. 10.1002/gps.1956 18229882

[B2] Carpenter B. D. Balsis S. Otilingam P. G. Hanson P. K. Gatz M. (2009). The Alzheimer’s disease knowledge scale: development and psychometric properties. Gerontologist. 49, 236–247. 10.1093/geront/gnp023 19363018 PMC2667675

[B3] Climent M. T. Pardo J. Muñoz-Almaraz F. J. Guerrero M. D. Moreno L. (2018). Decision tree for early detection of cognitive impairment by community pharmacists. Front. Pharmacol. 9, 1232. 10.3389/fphar.2018.01232 30420808 PMC6215965

[B4] Criddle D. (2014). The role of pharmacists in the early detection of dementia. Aust. Pharm. 33, 38–41.

[B5] Foreman K. Marquez N. Dolgert A. Fukutaki K. Fullman N. McGaughey M. (2018). Forecasting life expectancy, years of life lost, and all-cause and cause-specific mortality for 250 causes of death: reference and alternative scenarios for 2016–40 for 195 countries and territories. Lancet 392, 2052–2090. 10.1016/S0140-6736(18)31694-5 30340847 PMC6227505

[B6] Garre-Olmo J. (2018). Epidemiología de la enfermedad de Alzheimer y otras demencias. Rev. Neurol. 66 (11), 377–386. 10.33588/rn.6611.2017519 29790571

[B7] Hudson J. Pollux P. Mistry B. Hobson S. (2012). Beliefs about Alzheimer’s disease in Britain. Aging and Ment. Health 16, 828–835. 10.1080/13607863.2012.660620 22416945

[B8] Hughes M. L. Lowe D. A. Shine H. E. Carpenter B. D. Balsis S. (2015). Using the Alzheimer’s Association web site to improve knowledge of Alzheimer’s disease in health care providers. Am. J. Alzheimers Dis. Other Demen. 30 (1), 98–100. 10.1177/1533317514559827 25425736 PMC10852924

[B9] INE: Instituto Nacional de Estadística (2016). Defuncones según la Causa de Muerte. Madrid Available: https://www.ine.es/jaxi/Datos.htm?path=/t15/p417/a2016/l0/[amp]file=02001.px

[B10] Jorge C. Cetó M. Arias A. Blasco E. Gil M. P. López R. (2018). Level of understanding of Alzheimer disease among caregivers and the general population. Neurología. 10.1016/j.nrl.2018.03.004 34238525

[B11] Löffler C. Koudmani C. Böhmer F. Paschka S. D. Höck J. Drewelow E. (2017). Perceptions of interprofessional collaboration of general practitioners and community pharmacists - a qualitative study. BMC Health Serv. Res. 17, 224. 10.1186/s12913-017-2157-8 28327136 PMC5359890

[B12] Kivipelto M. Ngandu T. Fratiglioni L. Viitanen M. Kåreholt I. Winblad B. (2005). Obesity and vascular risk factors at midlife and the risk of dementia and alzheimer disease. Arch. Neurol. 62 (10), 1556–1560. 10.1001/archneur.62.10.1556 16216938

[B13] Mat Nuri T. H. Hong Y. H. Ming L. C. Mohd Joffry S. Othman M. F. Neoh C. F. (2017). Knowledge on Alzheimer's Disease among Public Hospitals and Health Clinics Pharmacists in the State of Selangor, Malaysia. Front. Pharmacol. 8:739. 10.3389/fphar.2017.00739 29123479 PMC5662870

[B14] Martínez-Lage P. Martín-Carrasco M. Arrieta E. Rodrigo J. Formiga F. (2018). Map of Alzheimer’s disease and other dementias in Spain. MapEA Project. Rev. Esp. Geriatr. Gerontol. 53 (1), 26–37. 10.1016/j.regg.2017.07.006 29107401

[B15] Millard F. Baune B. (2009). Dementia – who cares? A comparison of community needs and primary care services. Aust. Fam. Physician 38 (8), 642–649. https://www.ncbi.nlm.nih.gov/pubmed/19893788 19893788

[B16] Nepal H. Jeffrey B. Bhattarai M. (2017). Dementia: risk factors and updated review. J. Psychiatrists Assoc. Nepal 2, 3–7. 10.3126/jpan.v6i2.21750

[B17] Nordhus I. Sivertsen B. Pallesen S. (2012). Knowledge about alzheimer’s disease among norwegian psychologists: the alzheimer’s disease knowledge scale. Aging Ment. Health 16, 521–528. 10.1080/13607863.2011.628973 22129312

[B18] OECD/European Observatory on Health Systems and Policies (2017). Spain: Country Health Profile 2017, State of Health in the EU. Brussels: OECD Publishing, Paris/European Observatory on Health Systems and Policies. 10.1787/9789264283565-en

[B19] Perry M. Drašković I. Van Achterberg T. Borm G. F. Van Eijken M. I. Lucaassen P. (2008). Can an EASYcare based dementia training programme improve diagnostic assessment and management of dementia by general practitioners and primary care nurses? The design of a randomised controlled trial. BMC Health Serv. Res. 8, 71. 10.1186/1472-6963-8-71 18384675 PMC2391160

[B20] Prince M. J. Wimo A. Guerchet M. M. Ali G. C. Wu Y. T. Prina M., (2015). World Alzheimer Report 2015- The Global Impact of Dementia: An Analysis of Prevalence, Incidence, Cost and Trends. London: Alzheimer´s Disease Internacional.

[B21] Reis J. P. Loria C. M. Launer L. J. Sidney S. Liu K. Jacobs D. R. (2013). Cardiovascular health through young adulthood and cognitive functioning in midlife. Ann. Neurol. 73 (2), 170–179. 10.1002/ana.23836 23443990 PMC3608821

[B22] Santos C. Y. Snyder P. J. Wu W. C. Zhang M. Echeverria A. Alber J. (2017). Pathophysiologic relationship between Alzheimer’s disease, cerebrovascular disease, and cardiovascular risk: a review and synthesis. Alzheimers Dement. (Amst.) 7, 69–87. 10.1016/j.dadm.2017.01.005 28275702 PMC5328683

[B23] Santschi V. Chiolero A. Burnand B. Colosimo A. L. Paradis G. (2011). Impact of pharmacist care in the management of cardiovascular disease risk factors: a systematic review and meta-analysis of randomized trials. Arch. Intern. Med. 171, 1441–1453. 10.1001/archinternmed.2011.399 21911628

[B24] Skelton J. (2008). White paper on expanding the role of pharmacists in caring for individuals with Alzheimer’s disease: APhA Foundation Coordinating Council to Improve Collaboration in Supporting Patients with Alzheimer’s Disease Journal of the American Pharmacists Association. JAPhA 48, 715–721. 10.1331/JAPhA.2008.08144 19019799

[B25] Smyth W. Fielding E. Beattie E. Gardner A. Moyle W. Franklin S. (2013). MacAndrew MA survey-based study of knowledge of Alzheimer’s disease among health care staff. BMC Geriatr. 13, 2. 10.1186/1471-2318-13-2 23282030 PMC3543846

[B26] Stella F. Radanovic M. Canineu P. R. De Paula V. J. Forlenza O. V. (2015). Anti-dementia medications: current prescriptions in clinical practice and new agents in progress. Ther. Adv. Drug. Saf. 6 (4), 151–165. 10.1177/2042098615592116 26301069 PMC4530351

[B27] Steven C. 2015 World Dementia Council issues risk reduction statement. Global action against dementia. Alzheimer’s Association Statement.

[B28] Vernooij-Dassen M. J. Moniz-Cook E. D. Woods R. T. De Lepeleire J. Leuschner A. Zanetti O. (2005). Factors affecting timely recognition and diagnosis of dementia across Europe: from awareness to stigma. Int. J. Geriatr. Psychiatry 20, 377–386. 10.1002/gps.1302 15799080

[B29] World Health Organization (2007). Global age-friendly cities: a guide. (1):3–5. WHO Press, Geneva, Switzerland (http://www.who.int/ageing/publications/Global_age_friendly_cities_Guide_English.pdf).

[B30] World Health Organization Dementia, 2017 http://www.who.int/es/news-room/fact-sheets/detail/dementia.

[B31] Yaffe K. Vittinghoff E. Pletcher M. J. (2014). Early adult to midlife cardiovascular risk factor and cognitive function. Circulation 129 (15), 1560–1567. 10.1161/CIRCULATIONAHA.113.004798 24687777 PMC4700881

